# *Notes from the Field:* Toxic Leukoencephalopathy Associated with Tianeptine Misuse — California, 2017

**DOI:** 10.15585/mmwr.mm6727a5

**Published:** 2018-07-13

**Authors:** Robert Goodnough, Kai Li, Fatemeh Fouladkou, Kara L. Lynch, Maulik Shah, Craig G. Smollin, Paul D. Blanc

**Affiliations:** ^1^California Poison Control System San Francisco Division; ^2^Department of Emergency Medicine, University of California San Francisco; ^3^Department of Laboratory Medicine, University of California San Francisco; ^4^Department of Neurology, University of California San Francisco; ^5^Division of Occupational and Environmental Medicine, Department of Medicine, University of California San Francisco.

During the early morning of October 10, 2017, a California man aged 24 years was noted to be lethargic with slurred speech; at 2:30 p.m., he was found unresponsive. Emergency medical services transported him to an emergency department. The patient had a 2-year history of tianeptine misuse. Tianeptine is an atypical tricyclic antidepressant that enhances serotonin uptake, increases dopamine signaling, modulates glutamate signaling, and stimulates mu (*μ*) and delta (*δ*) opioid receptors (*1*,*2*). Tianeptine is taken for its anxiolytic, mood-enhancement, and euphoric effects (*3*). The patient had recent concomitant misuse of phenibut (β-Phenyl-γ-aminobutyric acid), a central nervous system depressant. Neither tianeptine nor phenibut is licensed in the United States; both were purchased online. The patient’s medical history included sleep apnea, depression, anxiety, and attention deficit hyperactivity disorder (treated with methylphenidate). He occasionally misused prescription benzodiazepines and opiates, reportedly taken from family members.

Upon hospitalization, the patient was comatose but with intact brainstem reflexes and was intubated because of a low respiratory rate. An initial urine toxicology screen was positive only for marijuana. Two days after admission, brain magnetic resonance imaging (MRI) indicated diffuse white matter damage characteristic of toxic leukoencephalopathy. The patient was transferred to a tertiary care facility. On October 15, repeat MRI imaging confirmed leukoencephalopathy involving almost the entire supratentorial white matter. The patient’s neurologic status deteriorated with development of prolonged extensor and flexor posturing and loss of brainstem reflexes; he died 19 days after his initial admission.

Serum from October 10 was tested for a range of exogenous substances by liquid chromatography–high resolution mass spectrometry. The tianeptine level was 3,000 ng/mL (therapeutic range = 278–366 ng/mL) (*3*); phenibut was undetectable. Benzodiazepines and their metabolites within therapeutic ranges included clonazepam, 7-aminoclonazepam, midazolam, and alpha-hydroxymidazolam. Also detected were the central nervous system stimulant methylphenidate; tetrahydrocannabinol (THC) (the psychoactive constituent of cannabis); and its metabolite, hydroxyl-THC. The comprehensive blood testing and the initial urine screen were negative for opiates. Given the role of tianeptine in this patient’s outcome, and its potential for public health impact, an adverse event report has been filed with the Food and Drug Administration.

Tianeptine overdose fatalities are associated with serum concentrations ranging from 4,000 to 18,000 ng/mL (*4*). Tianeptine dependence and a withdrawal syndrome of anxiety, sweating, myalgias, chills, and depression have been described (*2*). This is the first known case of toxic leukoencephalopathy reported associated with tianeptine. Toxic leukoencephalopathy can be distinguished from leukoencephalopathy associated with hypoxia by delayed onset and by radiographic features. Other illicit toxicants have been associated with acute toxic leukoencephalopathy, including inhalation of heroin combustion byproducts (“chasing the dragon”) (*5*). The patient’s tianeptine use, with a blood concentration an order of magnitude higher than therapeutic levels, implicates it in this patient’s acute illness and findings although this does not confirm causality. The absence of supratherapeutic levels of other pharmaceuticals reduces the likelihood that they directly led to leukoencephalopathy although drug interactions cannot be excluded as contributors. The negative urine and blood testing for opiates and the absence of a history of heroin inhalation make this an unlikely etiology for the leukoencephalopathy in this case. Other pharmaceuticals have been implicated in toxic leukoencephalopathy, further precluding any definitive etiological conclusion based on a single observation. Nevertheless, this case highlights the potential of tianeptine misuse to emerge as a public health issue, whether used alone or in the context of polysubstance use. Health care providers should be aware of tianeptine misuse, including its potential link to severe adverse outcomes.
